# Accelerating investigation of new HIV drugs in pregnancy: advancing the research agenda from theory to action

**DOI:** 10.1002/jia2.25912

**Published:** 2022-07-19

**Authors:** Martina Penazzato, Shahin Lockman, Angela Colbers, Françoise Renaud, Alexandra Calmy, Polly Clayden, Marissa Vicari, Imelda C. Mahaka, Jennifer M. Zech, Cadi Irvine, Elaine J. Abrams, the participants of the WHO‐IMPAACT Workshop “Approaches to Enhance and Accelerate Study of New Drugs for HIV and Associated Infections in Pregnant Women”

**Affiliations:** ^1^ Department of Global HIV Hepatitis and Sexually Transmitted Infections Programmes World Health Organization Geneva Switzerland; ^2^ Brigham and Women's Hospital Harvard TH Chan School of Public Health Boston Massachusetts USA; ^3^ Department of Pharmacy Radboud Institute for Health Sciences Radboud University Medical Center Nijmegen Netherlands; ^4^ HIV/AIDS Unit Division of Infectious Diseases Geneva University Hospitals Geneva Switzerland; ^5^ HIV i‐Base London United Kingdom; ^6^ HIV Programmes and Advocacy Department International AIDS Society Geneva Switzerland; ^7^ Pangaea Zimbabwe AIDS Trust (PZAT) Harare Zimbabwe; ^8^ ICAP at Columbia University New York City New York USA; ^9^ Vagelos College of Physicians and Surgeons Columbia University New York City New York USA

**Keywords:** ARV, clinical trials, gender, HIV prevention trials, treatment, women

## Abstract

**Introduction:**

Historical approaches to clinical development of novel therapeutics for treatment and prevention of HIV have led to unacceptable delays in the generation of data to support optimal antiretroviral drug use in pregnancy. Over the last 5 years, multiple stakeholders have voiced their concerns around the exclusion of pregnant women from drug trials, and some progress has been made to consolidate principles and forge consensus. Building on ongoing efforts, the World Health Organization (WHO) and the International Maternal Paediatric Adolescent AIDS Clinical Trials Network (IMPAACT) convened a technical consultation designed to move the discussion from theory to practice.

**Discussion:**

Accelerating the inclusion of pregnant women in pre‐licensure clinical trials, with a goal to have pharmacokinetics (PK) and preliminary safety data for all new HIV agents in pregnancy available at the time of drug approval, requires: (1) performing non‐clinical developmental and reproductive toxicology studies early in drug development for all new HIV agents; (2) recognizing and acting on the central role of women of childbearing potential affected by HIV through the research being conducted and the dissemination of associated results; (3) enrolling pregnant women in studies to specifically determine pregnancy PK and preliminary safety, as soon as late non‐clinical studies are completed with no negative signals, for all new HIV agents that have demonstrated preliminary evidence of safety and efficacy from phase 2 trials; (4) investigating adverse pregnancy and birth outcomes through dedicated pregnancy safety studies for all new priority HIV agents; and (5) expanding active surveillance of drug safety in pregnancy for rare events, such as birth defects.

Strategic actions to pursue include developing tools and resources to support designing and implementing studies among pregnant and breastfeeding women, identifying and promoting modifications of the regulatory framework that are supportive of systematic ethical investigation of new drugs in pregnancy, coordinating surveillance efforts, mobilizing key stakeholders and promoting transparency and accountability for all involved.

**Conclusions:**

With more than 19 million women living with HIV worldwide, ensuring greater inclusion of pregnant women in research on novel therapeutics is a priority to support drug optimization and effective introduction of innovations for treatment and prevention of HIV.

## 1 INTRODUCTION

Despite tremendous work by the research community in exploring effective strategies to prevent HIV vertical transmission, historical approaches to clinical development of novel therapeutics for treatment and prevention of HIV have led to unacceptable delays of up to a decade after initial drug approval in the generation of data to support optimal antiretroviral (ARV) drug use in pregnancy [1]. This has in turn not just excluded pregnant women from receiving new, optimized agents and regimens, but also impeded rapid rollout of new HIV treatment and prevention agents in settings where women of childbearing potential constitute the majority of people at risk of acquiring HIV or living with HIV [2].

Over the last 5 years, multiple stakeholders have voiced their concerns around the exclusion of pregnant women from drug trials and the associated harms and risks of these policies. As recently outlined by the PHASES group [3], a paradigm shift is now required to promote ethical inclusion rather than presumptive exclusion of pregnant women from clinical drug trials.

Some progress has been made in recent years to consolidate principles and forge consensus on the need to generate better data more rapidly on HIV agents for pregnant women. Building on ongoing efforts by regulators and the United States *Eunice Kennedy Shriver* National Institute of Child Health and Human Development Task Force on Research Specific to Pregnant Women and Lactating Women (PRGLAC), the WHO‐convened Paediatric ARV Drug Optimization (PADO) and Conference on ARV Drug Optimization (CADO) groups [4], and the International AIDS Society's Collaborative Initiative for Paediatric HIV Education and Research (CIPHER), the WHO, and the International Maternal Paediatric Adolescent AIDS Clinical Trials Network (IMPAACT) collectively convened two technical consultations to identify optimal approaches to studying the pharmacokinetics (PK), safety and efficacy of new HIV‐related agents during pregnancy [5, 6].

These consultations were designed to move the discussion from theory to practice and concretely outline how to make the paradigm shift a true change in practice for future impact. The first workshop, held in June 2019, reviewed how best to investigate PK in pregnant women [7]. The second workshop, “Approaches to Enhance and Accelerate Study of New Drugs for HIV and Associated Infections in Pregnant Women,” was launched virtually in December 2020, and brought together more than 100 participants from across the world, including women living with HIV, academic researchers, clinical experts, regulators, industry representatives, funders and other key stakeholders involved in studying drugs for the prevention and treatment of HIV and other infectious diseases among pregnant women. Through the WHO‐IMPAACT Workshop, the growing momentum towards this change was pushed forward with concrete actions to help take the field “from theory to practice.” The Workshop put the voice of women living with HIV at the centre of these discussions and identified core principles to enable an ethical shift towards greater inclusion of pregnant women as well as strategic actions that need to be urgently taken to ensure that this momentum is not lost. This paper reports the high‐level outcomes of the Workshop and serves as an introduction to other papers included in this supplement, which examine the technical discussions that occurred in more detail [6].

## 2 DISCUSSION

Many of the barriers to obtaining data on new agents in pregnancy can be attributed to historical approaches to drug development that focus on protecting the foetus from any potential harm. Delayed completion of pre‐clinical reproductive toxicology studies is one reason why pregnant women (or women who could become pregnant) have been excluded from pre‐licensure studies. It provides the rationale for requiring women who take part in clinical trials to agree to rigid contraception requirements and for discontinuing study drug if they become pregnant while in the trial [8]. This is particularly common when effective alternative options exist, and new therapeutic options may be perceived to have a marginal benefit on maternal and child outcomes. This reluctance is oftentimes fuelled by liability concerns and fear of litigation should exposure to the drug result in unintended consequences that may have life‐long implications. This has indeed affected the interest and motivation of sponsors, institutional review boards, regulatory agencies and researchers to conduct studies in pregnant women, resulting in their exclusion from pre‐licensure trials and long delays in obtaining critical PK and safety data [3, 9]. As a result, the PK and safety data that are available for ARV drugs when used during pregnancy are limited, and nearly always obtained through opportunistic, post‐marketing studies among women who become pregnant while using the new agent [10, 11].

While these practices are designed to protect the foetus from potential harm, they in fact do not entirely remove the risks for the foetus but rather shift them to the post‐marketing phase when women who are prescribed the new agent become pregnant and are then confronted with a complete lack of information, including basic dosing and safety during pregnancy to help decide whether to stay on the new agent or discontinue it.

This situation is not unique to HIV. In the United States, more than two in three women take at least one prescription medication during pregnancy [12], and 70% of medications approved by the US Food and Drug Administration (FDA) between 2000 and 2010 had no data around human pregnancy [13]. Today, the COVID‐19 pandemic continues to remind us of the importance of accelerating research during pregnancy. Surveillance of COVID‐19 vaccine safety during pregnancy was rapidly implemented by many stakeholders, an encouraging development. However, despite their significantly higher risk for poor COVID and birth outcomes, pregnant women were excluded from the vast majority of vaccine and therapeutic trials, leading to reluctance to use and prescribe these agents in pregnancy [14].

Inspiration can be drawn from the evolution of paediatric clinical development programmes and the improvements brought by the setup of the paediatric regulatory frameworks designed to overcome historical conservative approaches that resulted in lack of therapeutic options and delays in access to innovation across therapeutic areas. Despite some remaining delays, these frameworks now enable greater investigation of new drugs in children [15].

### 2.1 Keeping women's voice at the centre and enabling ethical shifts

The voices of women living with HIV are emerging loud and clear: action is needed to ensure that they are not left behind. Women want more information about the efficacy and safety of the drugs they are prescribed and more data to inform their providers’ advice regarding ARV treatment or prevention regimens. More information can only be generated if women are allowed to participate in clinical trials and benefit from the innovation brought by these studies. Women also expressed frustration at being required to use contraceptive measures they might not be willing to take in order to take part in clinical trials. It is also important for women to have the opportunity to decide whether or not to stay on study drug if they become pregnant in a clinical trial, with informed consent following discussion of potential risks, benefits and unknowns [16].

Women living with HIV should not be merely considered study participants, but should be involved in all aspects of research of new ARVs for HIV treatment and prevention [17]. Women from affected communities should be involved in the prioritization process as agents are identified for study in pregnancy, in the design, recruitment and implementation of studies, and in the interpretation and dissemination of study results. Establishing dedicated community advisory boards with the appropriate representation of women living with or at risk of HIV could be a practical step to ensure each clinical development programme benefits from the input of the community. Including women in the development process is expected to enhance all aspects of research and accelerate the study of novel HIV agents in pregnancy.

### 2.2 Approaches to expedite the timeline for the study of new ARVs in pregnancy

Current guidance recommends that all nonclinical toxicity studies assessing effects on female reproduction and development of the embryo, foetus and offspring, as well as the standard battery of genotoxicity tests, be completed prior to the inclusion of women of childbearing potential not using highly effective contraception, those whose pregnancy status is unknown and pregnant women in pre‐licensure trials [8]. Accelerating the completion of these nonclinical developmental and reproductive toxicology (DART) studies will be essential for the earlier inclusion of pregnant women in pre‐licensure trials. Ideally, fertility and early embryonic development (FEED) and embryo‐foetal development (EFD) studies should be completed during or no later than the end of the phase 2 registrational trial conducted in the general population [1]. The more complex and costly nonclinical pre‐ and postnatal development (PPND) studies should be completed during early phase 3 or no later than the end of the phase 3 registrational trial. Furthermore, complementary strategies, such as alternative combinations of DART study designs and physiologically based PK modelling, could better inform drug dosing and safety in pregnancy at an earlier stage in drug development, supporting earlier inclusion of pregnant women in clinical trials.

Assuming that DART studies will be completed earlier than current practice, once the non‐clinical FEED and EFD studies are completed (if no concerning signals are detected) and drug dosing has been established in non‐pregnant people, women who become pregnant in registrational trials should be given the option to make an informed choice as to whether or not they wish to stay in a phase 3 trial of a new agent. Similarly, once both the FEED and PPND studies are completed, with no negative signals (and dosing established in non‐pregnant people), pregnant women can be enrolled in specific studies to determine dosing and preliminary safety. Ideally, dosing in pregnancy would be determined for all new agents for HIV prevention and treatment in time to be included in the drug label detailing the regulatory approval.

These decisions would need to incorporate accurate considerations on the potential risk and benefit balance. The risks of exposure to the new agents are anticipated to be different for each of the three trimesters of pregnancy, with exposure during the first posing the greatest risk for organogenesis, and during the third being of lesser concern for teratogenicity but a period during which foetal growth and birth outcomes can be affected. Assessing the benefits also requires examining the degree of unmet need with instances where no other therapeutic options exist (e.g. in the presence of a multiclass resistant virus) with a clear added benefit in contrast with situations in which effective alternative options are available and fewer benefits may be associated with the use of the new agent (e.g. comparing a long‐acting pre‐exposure prophylaxis agent vs. standard oral daily regimens). These scenarios should be taken into consideration by researchers, regulators, clinicians and women involved with designing, approving and participating in the trial.

Similar to most recent approaches for paediatric clinical development, new HIV treatment agents that are demonstrated to be efficacious in non‐pregnant adults, defined by viral load suppression, do not need to be studied for efficacy in pregnancy as long as PK is comparable and appropriate dosing is established [18]. In addition, if adequate drug exposures are achieved in pregnancy, efficacy for prevention of vertical transmission does not need to be studied directly and can be inferred. However, it is critical that new priority HIV agents that are likely to be of public health importance for young women be investigated for adverse maternal, pregnancy and birth outcomes through dedicated pregnancy safety studies as soon as dosing is confirmed. Key outcomes for pregnancy safety trials include birth outcomes (prematurity, small for gestational age, spontaneous abortion and stillbirth) and neonatal death.

Recognizing that current approaches to study design often contribute to long delays in obtaining critical pregnancy data for new agents, alternatives are needed to the typical stand‐alone, sequential randomized clinical trials comparing a new agent to the standard of care. A number of approaches to Master Protocols (such as platform trials, adaptive trials or basket trials) have received support and guidance from the FDA and have the potential to produce relevant data more quickly [19]. Platform trials may be the most applicable approach to studying safety in pregnancy due to their multi‐arm nature and the possibility to add new agents as they become available [19, 20].

Concern about teratogenicity risk of a new agent is an important driver of the protective framework that leads to the exclusion of pregnant women from pre‐licensure trials. However, detection of congenital anomalies requires the evaluation of large numbers of early pregnancy exposures, which can only be collected through post‐marketing studies. Furthermore, more data on birth outcomes in non‐study settings are essential to fully assess safety of any new agent in pregnancy. Therefore, it is critical that rigorous safety surveillance studies are conducted to ensure the systematic and rapid detection of adverse birth outcomes and rare events, such as birth defects associated with exposure to ARVs during pregnancy. A new collaborative conceptual framework for active surveillance of ARV safety in pregnancy can enhance this surveillance by emphasizing data harmonization and standardization of outcomes between surveillance programs, sharing materials and tools, use of innovative approaches and creating linkages and collaboration between programs [21].

### 2.3 Strategic actions to implement a new framework for investigating new drugs for HIV prevention and treatment for pregnant and breastfeeding women

Theory can turn into practice only when action is taken to concretely accelerate the generation of more and better data on new HIV agents in pregnancy. A number of actions, summarized in Table 1, have been prioritized to better target joint efforts and prompt a concrete shift in practice by providing tools, guidance and resources as examples and templates for those involved in designing and implementing studies on new HIV agents; by spurring further early‐stage research to inform future modifications and the regulatory framework; by generating alignment and fostering coordination among those contributing to surveillance efforts; by sharing knowledge and mobilizing key stakeholders through targeted advocacy; and by promoting transparency and accountability for all stakeholders involved.

**Table 1:** Strategic actions to accelerate generation of data for new HIV agents in pregnancy

Share and confirm proposed timing of developmental and reproductive toxicology with stakeholders, including industry, regulatory agencies and national authorities. Define optimal approaches to communicate non‐clinical developmental and reproductive toxicology study results into clinical and lay language for use in clinical trial materials (patient information sheets and investigator brochures) to ensure better understanding by clinical trial participants of the non‐clinical study results and how these results informed the clinical trial. Provide support to and make resources available to institutional review boards and ethics committees to review and interpret DART study results. Support innovation of reproductive toxicology studies to enable earlier inclusion of pregnant women in clinical trials through several mechanisms: promote (improving and validating) physiologically based pharmacokinetic modelling to support drug development and inform the dose selected for use among pregnant women; encourage alternative strategies, such as enhanced and combination studies that include both embryo‐foetal development and pre‐ and postnatal development studies to move studies earlier and complete studies more rapidly to enable pregnant women to be included in phase 3 trials; and support scientific research to advance alternatives and supplements to current approaches to non‐clinical studies. Examine how clinical trials insurance and liability requirement impact inclusion of pregnant women in trials. Adopt harmonized definitions of adverse pregnancy, birth and maternal outcomes and establishing basic (minimum) agreed safety endpoints to collect in trials involving pregnant women and surveillance studies to enable harmonization and data sharing across studies. Develop a toolkit for research in pregnancy in support of investigators and key stakeholders as well as an inventory to include: study protocols that represent good practices of implementation of core principles; simplified template case report forms for pregnancy studies that capture the essential exposure and outcome information to facilitate efficiency; and template protocols for open access and wide distribution, to support design of staged enrolment (first enrolling women in their late third trimester followed by enrolments in the early third trimester and finally enrolling women during the second trimester) and integrated pregnancy pharmacokinetic and pregnancy safety trial. Identify and engage with existing country programmes and safety surveillance studies in pregnancy for central sharing and standardization. Identify 3–5 existing or new sites to implement a modular format for active surveillance of the safety of HIV drugs in pregnancy (with denominators) for the key outcomes. Monitor progress and promote accountability of key stakeholders: create targets and monitoring and evaluation goals for monitoring progress on implementation of above strategic actions; establish a forum for achieving these aims and for facilitating ongoing discussions related to study design and implementation across stakeholder groups; advocate for funding mobilization; and advocate for guidance from regulatory agencies and key stakeholders.

Note: The conclusions presented in this table are drawn from the WHO/IMPAACT Meeting Report on Approaches to enhance and accelerate study of new drugs for HIV and associated infections in pregnant women [6].

## 3 CONCLUSIONS

With more than 19 million women living with HIV worldwide and 600,000 women with newly acquired HIV infections each year [22], ensuring greater inclusion of pregnant women in research on novel therapeutics is a priority to support drug optimization and effective introduction of innovations for treatment and prevention of HIV.

Findings from the Workshop were broadly disseminated through a number of conferences [23, 24, 25] and fed into the WHO drug optimization process undertaken during the CADO4 meeting [26], where the core principles were applied to identify key research priorities for pregnant women. This article, together with the other articles of this supplement, provides deeper understanding of some of the barriers and proposed solutions.

While the outcomes of this process advocate for inclusion of pregnant women in clinical trials investigating new HIV agents used for treatment or prevention of HIV, they can also be applied to HIV‐associated infections and potentially adapted for other conditions that impact the health of women of reproductive potential. Leveraging the work of the HIV community may help those working in other disease areas to accelerate a similar shift in thinking and practice, which should be urgently pursued.

These collective efforts are a testimony of the commitment of WHO, the IMPAACT network and others in leveraging their convening power and research expertise to rally a movement made of many stakeholders who all have a central role to play to ensure a successful transition from theory to practice.

## COMPETING INTERESTS

AC received research grants to their institution from Merck, Gilead and ViiV Healthcare.

## AUTHORS’ CONTRIBUTIONS

MP and EJA developed the first draft of the manuscript and finalized it after contributions from other co‐authors. All authors have read and approved the final manuscript.

## Data Availability

The views expressed in this article are the personal views of the author(s) and may not be understood or quoted as being made on behalf of or reflecting the position of the regulatory agency/agencies or organizations with which the author(s) is/are employed/affiliated.
